# Assessing clinicians’ Post-Exposure Prophylaxis recommendations for rabies virus exposures in Hunan Province, China

**DOI:** 10.1371/journal.pntd.0009564

**Published:** 2021-07-06

**Authors:** Yu Li, Jeanette J. Rainey, Hao Yang, Cuc H. Tran, Yang Huai, Rongqiang Liu, Hongwei Zhu, Zhengliang Wang, Di Mu, Wenwu Yin, Chun Guo, Miriam Shiferaw, Qiulan Chen, Shixiong Hu, Zhongjie Li

**Affiliations:** 1 Division of Infectious Disease, Key Laboratory of Surveillance and Early-Warning on Infectious Disease, Chinese Center for Disease Control and Prevention, Beijing, China; 2 Division of Global Health Protection, United States Centers for Disease Control and Prevention, Beijing, China; 3 Hunan Province Center for Disease Control and Prevention, Changsha, China; 4 Division of High Consequence Pathogens and Pathology, United States Centers for Disease Control and Prevention, Atlanta, Georgia, United States of America; 5 Shuangfeng Center for Disease Control and Prevention, Shuangfeng, China; 6 School of Public Health, Huazhong University of Science and Technology, Wuhan, China; BCG Vaccine Laboratory, INDIA

## Abstract

**Background:**

Timely and appropriate administration of post-exposure prophylaxis (PEP) is an essential component of human rabies prevention programs. We evaluated patient care at rabies clinics in a high-risk county in Hunan Province, China to inform strategies needed to achieve dog-mediated human rabies elimination by 2030.

**Methods:**

We collected information on PEP, staff capacity, and service availability at the 17 rabies clinics in the high-risk county during onsite visits and key staff interviews. Additionally, we conducted observational assessments at five of these clinics, identified through purposive sampling to capture real-time information on patient care during a four-week period. Wound categories assigned by trained observers were considered accurate per national guidelines for comparison purposes. We used the kappa statistic and an alpha level of 0.05 to assess agreement between observers and clinic staff.

**Results:**

In 2015, the 17 clinics provided PEP to 5,261 patients. Although rabies vaccines were available at all 17 clinics, rabies immune globulin (RIG) was only available at the single urban clinic in the county. During the assessment period in 2016, 196 patients sought care for possible rabies virus exposures. According to observers, 88 (44%) patients had category III wounds, 104 (53%) had category II wounds and 4 (2%) had category I wounds. Observers and PEP clinic staff agreed on approximately half of the assigned wound categories (kappa = 0.55, p-value< 0.001). Agreement for the urban county-level CDC clinic (kappa = 0.93, p-value<0.001) was higher than for the township clinics (kappa = 0.16, p-value = 0.007). Using observer assigned wound categories, 142 (73%) patients received rabies vaccinations and RIG as outlined in the national guidelines.

**Conclusion:**

Rabies PEP services were available at each town of the project county; however, gaps between clinical practice and national rabies guidelines on the use of PEP were identified. We used these findings to develop and implement a training to rabies clinic staff on wound categorization, wound care, and appropriate use of PEP. Additional risk-based approaches for evaluating human rabies virus exposures may be needed as China progresses towards elimination.

## Author summary

Members of the United Nations agreed on a goal of eliminating dog-mediated rabies by 2030. To achieve this goal, China and other endemic rabies countries will need to increase dog rabies vaccination coverage as well as ensure proper administration of rabies post-exposure prophylaxis (PEP) for patients with animal wounds. PEP includes wound washing, timely vaccination, and rabies immune globulin (RIG) according to the type and severity of patients’ wounds. The authors conducted an observational assessment of PEP clinics in a high-risk county in Hunan Province. Most patients at these clinics received PEP according to the national guidelines; however, certain patients received PEP, when not warranted, and others did not receive RIG, as recommended for severe animal wounds. Wound type categorization and corresponding use of PEP were most accurate at the single urban clinic and least accurate at the rural clinics in the project county. RIG was only available at the single urban clinic. These findings highlight the importance of improving the use of PEP and access to RIG, particularly in rural areas, and exploring the use of risk-based approaches for evaluating human rabies virus exposures. These steps can contribute to eliminating dog-mediated rabies in the project county as well as elsewhere in China.

## Introduction

Rabies is a fatal zoonotic disease that causes more than 59,000 human rabies deaths each year. Almost 4 million people in Africa and Asia are currently at-risk of rabies, primarily through dog-mediated rabies virus exposures [1, 2]. In 2015, member states of the United Nations agreed on a goal of eliminating dog-mediated rabies by 2030. The World Health Organization (WHO), the World Organization for Animal Health (OIE), the Food and Agriculture Organization (FAO), and the Global Alliance for Rabies Control (GARC) developed a blueprint to increase the evidence-base and political commitment needed to achieve this goal [3]. Although effective dog rabies vaccination programs are essential for eliminating dog-mediated rabies, access to and appropriate use of post-exposure prophylaxis (PEP) will remain critical for preventing unnecessary human deaths [4].

Rabies PEP includes wound washing, timely PEP vaccination, and rabies immune globulin (RIG) for cases with transdermal and mucosal exposures. Health care workers at rabies clinics are required to apply complex vaccine regimens to various types of animal wounds, resulting in either overuse of PEP or missed opportunities to vaccinate, particularly for RIG [5]. The cost of PEP may be prohibitive, and access can be limited in certain areas due to procurement and distribution constraints, particularly in rural communities [6–8]. The integrated bite case management (IBCM) approach for assessing potential rabies virus exposures relies on assessing patient risk according to surveillance data on rabid dogs and timely investigation and follow-up of the wound event and biting animal prior to administering PEP and RIG [9]. This approach could be implemented to improve the appropriate use of PEP for patients seeking care for animal wounds in both rabies free and rabies endemic countries [6, 9–11]. The approach could increase the PEP supply and reduce the direct and indirect (e.g., time away from work) costs for patients.

In China, national public health officials are committed to achieving dog-mediated human rabies elimination by 2030. Since peaking in 2007 with more than 3,000 reported human rabies deaths, substantial progress has been made in reducing these deaths. Fewer than 500 human deaths were reported in 2018 [12]. The majority of these human rabies deaths occur among the young and elderly population residing in rural communities, and almost 85% are associated with wounds from rabid dogs [12]. Health care for animal wounds, including PEP, is available at county-level Centers for Disease Control (CDC) and township-based rabies clinics. These clinics, which are located in hospitals or vaccination centers, offer wound washing and PEP services according to the national guidelines for human rabies prevention and control [13].

In this project, we conducted an assessment of rabies clinics in a high-risk county in Hunan Province. We hypothesized that all clinics in the county were following the national guidelines for wound categorization and use of PEP following a rabies virus exposure. In addition to identifying opportunities to improve patient care, findings from the assessment can be used to determine the feasibility of introducing an integrated bite case management approach in Hunan and other high-risk provinces as part of China’s effort to achieve human rabies elimination.

## Methods

### Ethics statement

This public health evaluation project relied on the analysis of de-identified data. Verbal consent was obtained from participating clinic staff. No personal identifying information was recorded in the assessment tool. The Institutional Review Board (IRB) of Hunan CDC reviewed and approved the project protocol (S1 File) prior to implementation (IRB #2016009). The US CDC approved the project protocol as a program evaluation activity.

### Project location

**Fig 1:**
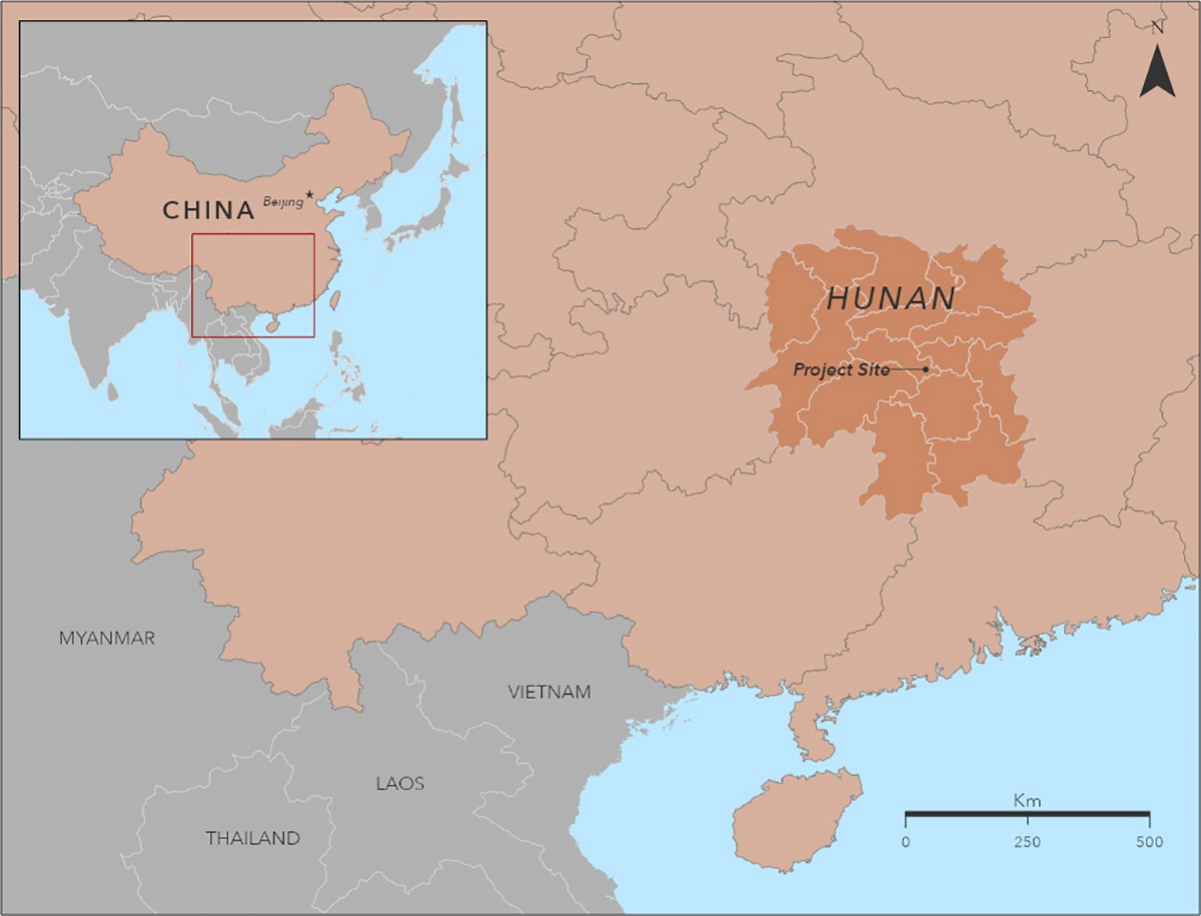
Location of rabies PEP project county A, Hunan Province, China, 2016. Project county is one of 122 counties in Hunan Province. The base layer of the map is from Natural Earth (https://www.naturalearthdata.com/).

Hunan Province is located in Central China (Fig 1) and is one of the five high-risk provinces for rabies in the country. Between 2011 and 2015, Hunan CDC reported 503 human cases of rabies. We used purposeful sampling to select a single county in the province for this project—county A. County A is located in one of the three high-risk prefectures for rabies in Hunan Province. The county CDC maintained a strong rabies surveillance unit (reported complete human rabies case investigation data to the National Notifiable Disease Reporting System [NNDRS] from 2004–2015), and county leadership expressed a commitment to the project. In 2016, the population of county A was approximately 879,000; 68% of this population resided in rural communities. The mean annual income for urban and rural residents was $2,681 and $1,448, respectively. Between 2004 and 2016, the county CDC reported 20 human rabies cases to NNDRS.

### National guidelines on human rabies prevention and control

According to the national guidelines in China, patients presenting with category II or III animal wounds should receive wound care and initiate rabies vaccination as soon as possible following possible rabies virus exposure (S1 Table). Patients with category III wounds should also receive RIG. Rabies vaccination series can be administered as either Zagreb 2-1-1, in which two doses of vaccine are injected intramuscularly on day 0 (one into each of the two deltoid or thigh sites) followed by one of each dose on days 7 and 21, or the five-dose Essen regimen, in which a single dose is administered intramuscularly on days 0, 3, 7, 14, and 28 [14]. The availability of each series (Zagreb or Essen) varies by clinic, and the procurement of which vaccine brand is determined by the clinic. Both human plasma (hRIG) and equine plasma (eRIG) rabies immune globulin are approved in China. All rabies clinics in the country follow the national guidelines. Patients typically pay for rabies PEP as an ‘out of pocket’ expense. Patients can request a partial reimbursement of this expense through the national health insurance program.

### Rabies clinic survey

We conducted a survey to describe clinic practices, vaccine management, and services offered at the 17 rabies clinics in county A. Three two-member teams conducted the clinic visits in June of 2016; each team was responsible for completing the survey at five to six clinics. A standard paper survey was used to collect information on clinic working hours and services provided (wound treatment, vaccination, and RIG), type and number of clinic staff, rabies PEP related equipment and drugs. Team members assessed vaccine management through visual inspection and by interviewing clinic staff. Team members also collected information from patient log forms at each clinic on the types of animal wounds treated, wound categories, wound care, vaccines, and RIG administered during 2015. Survey and log data were entered into an EpiData (version 3.1) database for cleaning and verification.

### Observational assessment

We selected five rabies clinics through purposive sampling for our observational assessment. The sample included the single urban county-level CDC clinic and the four rural-based township clinics with the most patients seeking care for animal wounds in 2015. The selected clinics were otherwise similar to the remaining 12 clinics in the county (S2 Table). Three two-member teams visited one to two of the five selected clinics during normal working hours to observe and record information on the demographic characteristics of each patient, wound description and care, and the wound category (I, II, or III) assigned by clinic staff and PEP provided to the patient.

These observational data were captured in real-time using a tablet-based collection tool. The tool, developed by China CDC, provided an online data entry screen, validation checks and automated skip patterns, and data editing functions. One team member entered the data in the tablet-based collection tool and the other member reviewed and verified the data record. Once internet service was available, data were automatically transmitted and saved on a China CDC server in Beijing. Data were reviewed daily by staff of Hunan CDC and edits and corrections were made directly in project database via the tablet data edit function.

Each project staff member was provided a tablet and training on the data collection tool as well as on data entry, editing, and data transmission to China CDC server. Rabies experts with Hunan CDC organized and conducted a training for project staff on wound categorization and PEP according to the national guidelines for human rabies prevention and control [13]. Each project staff member was required to pass a post-training examination that included reviewing and correctly categorizing photo images of 30 different types of animal wounds. The observational assessment was conducted from July 20 to August 5 in 2016, corresponding to the peak season for rabies virus exposures due to warmer temperatures and farm work (more time outdoors) [15]. Data were downloaded into an Excel database for cleaning and verification.

### Analysis

Our clinic survey and observational assessment data were imported into R version 3.5.1 (R Foundation for Statistical Computing, Vienna, Austria) for analysis. We described service hours, location, staff training, vaccine availability and management for each of the 17 rabies clinics in county A, stratifying by urban and rural setting. For the observational assessment, we generated descriptive statistics for patient demographics, wound category, and PEP provided during the clinic visit. We compared wound categories assigned by clinic staff with those made by team observers and generated an inter-rater kappa agreement statistic to quantify accuracy. We assumed that wound categories recorded by observers were accurate per national guidelines for the purpose of this comparison (based on the pre-assessment training). We estimated the percentage of patients receiving the correct type of PEP using wound categories assigned by clinic staff and by the team observers, respectively. An alpha level of 0.05 was used to assess statistical significance.

## Results

### Rabies clinic survey

**Fig 2:**
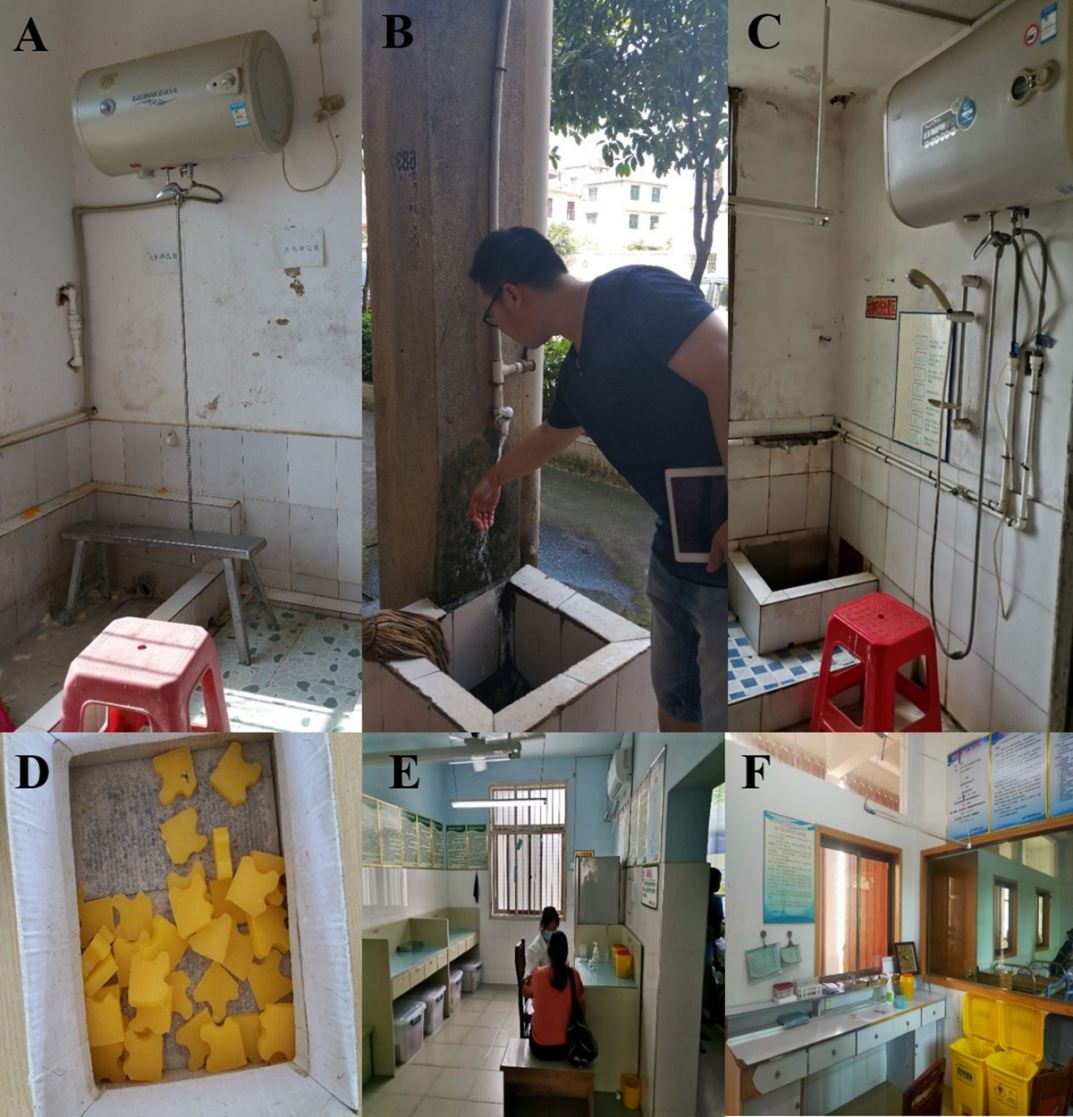
Snapshots from rabies PEP clinics in project county A, Hunan Province, China, July–August, 2016. (A) Facility for wound washing at the urban clinic. (B) Facility for wound washing at a rural clinic. (C) Facility for wound washing at a second rural clinic. (D) Soap used for wound washing at a rural clinic. (E) Area for vaccination and RIG administration at the urban clinic. (F) Area for vaccination at a rural clinic.

During 2015, the 17 clinics in county A provided PEP to 5,261 patients with possible rabies virus exposures (range by clinic: 0–1,954). Of the 17 clinics, 14 (82%) provided services 24 hours a day, 7 days a week (**Table 1**). The remaining three clinics (18%) provided services on weekdays and offered on-call services outside business hours. The educational level of staff at the urban CDC clinic was generally higher than that of staff at the rural township clinics; however, all staff had received previous training on rabies wound categories and PEP. All county clinics were using the five-dose Essen PEP vaccine series at the time of the project. The county CDC clinic provided RIG; other clinics did not. Rabies vaccines and RIG were maintained in cold storage as recommended in the national guidelines (i.e., refrigerated at 2–8°C). In 2016, the median cost for each rabies vaccination dose was US$10 (range by clinic: $9 - $12); completing the rabies vaccination series and a single dose of RIG (at the county CDC clinic) were US$48 (range by clinic: $43 - $61) and US$37, respectively. The clinic director or administrator established the cost for each PEP dose. In 2016, patients in county A could request a partial reimbursement (~US $25) for the rabies vaccination from the national insurance program. Nine clinics (53%) had special areas for wound treatment; none had professional wound treatment equipment (**Table 1 and Fig 2**). No human rabies cases were reported by county A in 2015 and 2016.

**Table 1:** Characteristics of 17 rabies clinics in project county A, Hunan Province, China, 2016.

Characteristics	Urban clinic (County CDC, N = 1)	Rural clinic (Township clinics, N = 16)	Total (N = 17)
**Number of patients seeking rabies PEP in 2015**	1,954	3,307	5,261
**Location setting, n (%)**			
** Urban**	1 (100)	0 (0)	1 (6)
** Rural**	0 (100)	16 (100)	16 (94)
Service hours of clinic^*^, n (%)			
** 24/7**	0 (100)	14 (88)	14 (82)
** Specific time of every day**	1 (100)	2 (13)	3 (18)
**Staff number per clinic, median [range]**	6 [6–6]	3.5 [1–7]	4 [1–7]
**Highest education level of staff, n (%)**			
** College**	2 (33)	2 (3)	4 (6)
** Junior college**	4 (67)	27 (42)	31 (44)
** Professional high school**	0 (0)	35 (55)	35 (50)
**With special area for wound treatment, n (%)**	1 (100)	8 (50)	9 (53)
**With professional wound washing equipment, n (%)**	0 (0)	0 (0)	0 (0)
**With refrigerator, n (%)**	1 (100)	16 (100)	17 (100)
**With rabies immunoglobulin, n (%)**	1 (100)	0 (0)	1 (6)
**Cost in US dollars for vaccine per whole course, median [range]**	48 [48–48]	48 [43–61]	48 [43–61]
**Cost in US dollars for rabies immunoglobulin per 200IU vial**^**†**^	37	--	37

^*^Service hours are determined separately by each PEP clinic.

^**†**^Rabies immunoglobulin derived from human blood (hRIG); hRIG is indicated for all transdermal and mucosal rabies virus exposures and is infiltrated around the wound site according to national PEP guidelines.

### Observational assessment

Team members observed care for 196 patients presenting with animal wounds at the five selected clinics (range by clinic: 15–111) (Table 2). Of these patients, 108 (55%) were male, 33 (17%) were 60 years of age or older and 83 (42%) were less than 18 years of age. Clinic staff obtained information on the source of the animal wound from almost all patients (n = 195, 99%). Of these patients, 140 had experienced dog bites or scratches. Less than 5% of these patients, however, were questioned about the dog’s health and vaccination status.

**Table 2:** Characteristics of patients seeking post-exposure prophylaxis (PEP) care at five selected rabies clinics in county A, Hunan Province, China, July–August of 2016.

Characteristics	Clinic 1 (county CDC, N = 111) n (%)	Clinic 2–5 (township hospitals, N = 85) n (%)	Total (N = 196) n (%)
**Sex**			
** Male**	54 (48)	54 (64)	108 (55)
** Female**	57 (51)	31 (36)	88 (45)
**Age group in years**			
** 0–5**	19 (17)	13 (15)	32 (16)
** 6–17**	24 (22)	27 (32)	51 (26)
** 18–60**	49 (44)	31 (36)	80 (41)
** > 60**	19 (17)	14 (16)	33 (17)
**Animal involved**			
** Dog**	84 (76)	56 (66)	140 (71)
** Cat**	18 (16)	20 (24)	38 (19)
** Rat** ^*^	9 (8)	7 (8)	16 (8)
** Human**	0 (0)	1 (1)	1 (1)
** Unknown**	0 (0)	1 (1)	1 (1)
**Animal ownership**			
** Self-owned**	44 (40)	49 (58)	93 (48)
** Neighbor-owned**	21 (19)	8 (9)	29 (15)
** Stray**	0 (0)	1 (1)	1 (1)
** Uncertain**	14 (17)	4 (5)	18 (9)
** No inquiry by clinic staff**	32 (29)	23 (27)	55 (28)
**Animal health status**			
** Healthy**	0 (0)	0 (0)	0 (0)
** Abnormal**	2 (2)	0 (0)	2 (1)
** Uncertain**	2 (2)	3 (4)	5 (3)
** No inquiry by clinic staff**	107 (96)	82 (96)	189 (96)
**Animal rabies vaccination history**			
** Vaccinated**	1 (1)	0 (0)	1 (1)
** Unvaccinated**	0 (0)	2 (2)	2 (1)
** No inquiry by clinic staff**	110 (99)	83 (98)	193 (98)

* There is currently no evidence to support rats as a source of human rabies. PEP was provided by clinic staff for these wound types based on patient’s request.

Of the 196 patients presenting with animal wounds, clinic staff categorized 51 (26%) with Type III wounds, 131 (67%) with Type II wounds, and 14 (7%) with Type I wounds. For these same patients, project observers categorized 88 (44%) with Type III wounds, 104 (53%) with Type II wounds and 4 (2%) with Type I wounds. Observers and PEP clinic staff agreed on approximately half of the assigned wound categories (kappa = 0.55, p-value <0.001). Agreement for the urban county-level CDC clinic (kappa = 0.93, p-value <0.001) was higher than for the rural township clinics (kappa = 0.16, p-value = 0.007) (Table 3). Poor agreement was statistically more likely to occur for patients presenting with wounds to hands and legs (27%) than other parts of the body (5%) (χ^2^ = 4.19, p-value = 0.041). Additionally, project observers were more likely than clinic staff to assign a patient with a Type III wound (χ^2^ = 18.51, p-value<0.001).

**Table 3:** Wound categories assigned by clinic staff and project observers for 196 patients seeking care for animal wounds and possible rabies virus exposures at five selected rabies clinics in county A, Hunan Province, China, 2016.

	Total, n (%)		Urban, n (%)		Rural, n (%)	
Wound Category *	Clinic Staff	Project Observers	Clinic Staff	Project Observers	Clinic Staff	Project Observers
** I**	14 (7)	4 (2)	0 (0)	2 (2)	14 (16)	2 (2)
** II**	131 (67)	104 (53)	61 (55)	57 (51)	70 (82)	47 (55)
** III**	51 (26)	88 (44)	50 (45)	52 (47)	1 (1)	36 (42)
**Total**	196	196	111	111	85	85

* Wound categories assigned by trained project observers were considered accurate and consistent with national guidelines for comparison purposes. Clinic staff and project observers agreed on approximately half of the assigned wound categories (kappa statistic = 0.55, p-value<0.001). Agreement was highest for the single urban clinic (kappa = 0.93, p-value<0.001) and lowest for the four rural clinics (kappa = 0.16, p-value = 0.007).

Of the 196 patients at the selected clinics, one patient at a rural clinic was transferred directly to the county CDC and excluded from the analysis. The remaining 195 patients received the rabies vaccination and 38 patients also received RIG. Using observer assigned wound categories, four patients with category I wounds unnecessarily received the rabies vaccination, while 49 patients with category III wounds should have received RIG but did not (Table 4). We monitored the human rabies surveillance system for reports from county A. According to the surveillance system, none of the patients observed during the PEP assessment developed rabies.

**Table 4:** Comparison of rabies post-exposure prophylaxis (PEP) recommended by national guidelines with actual rabies PEP received by 195 patients* at five selected clinics in county A, Hunan Province, China, July-August 2016.

Wound category ^†^	Wound Treatment n (%)		Rabies vaccine n (%)	Rabies immune globulin (RIG) n (%)
	Washing	Disinfection		
**Category I (N = 4)**	3 (75)	0 (0)	4 (100)	0 (0)
**Category II (N = 104)**	103 (99)	66 (63)	104 (100)	0 (0)
**Category III (N = 87)**	87 (100)	64 (74)	87 (100)	38 (44) ^‡^

^*^Of the 196 patients observed at the selected clinics, one patient at a rural clinic was transferred directly to the county CDC, and the remaining 195 patients were included in the analysis on PEP and RIG.

^**†**^Wound categories assigned by project observers were considered accurate and consistent with national guidelines for comparison purposes.

^‡^Apart from the 38 patients who received RIG as recommended, there were 49 patients (49/87, 56%) with category III wounds who did not receive RIG as recommended, including 13 patients (13/87, 15%) who were prescribed but refused RIG, and 36 patients (36/87, 41%) who were not prescribed RIG.

## Discussion

We reviewed practices at 17 rabies clinics in county A, Hunan Province and conducted an observational assessment at five clinics during the peak season for rabies virus exposures. Although rabies vaccine was available at all 17 clinics, RIG was only available at the urban clinic. Clinic staff had received previous training, and available vaccines were stored according to guidelines. Although most patients received PEP vaccination according to national guidelines, 49 patients did not receive RIG as recommended. Four additional patients unnecessarily received PEP for category I wounds. Most patients experienced dog bites or scratches; however, follow-up on the health and vaccination status of these dogs was limited. Based on these findings, we developed a training package for staff on wound care, wound categorization (focusing on wounds to the limbs), appropriate use of PEP, and increasing access to RIG for rural-based clinics. Hunan CDC has used this package to train PEP clinic staff in county A and elsewhere in the province. Introduction of an integrated risk-based bite management approach is feasible in county A and could further improve appropriate use of PEP and help reduce human rabies deaths [5, 6, 9–11].

Correct categorization of possible rabies virus exposures is necessary for appropriate wound treatment as well as rabies vaccination and RIG administration. Our assessment suggested a gap between clinical practice and national human rabies prevention and control guidelines. In general, clinical staff were more likely to under-diagnose, and by consequence, under-prescribe rabies RIG for patients with category III wounds when compared to project observers, particularly for patients seen at the rural township clinics. This may have been partially driven by the lack of access to RIG in rural clinics. Our finding is consistent with previous work highlighting urban-rural disparity in access to health care in China [16–18] and other rabies endemic countries [6, 8, 10]. Fewer training opportunities for staff, poorly equipped facilities, and greater financial barriers (e.g., lack of pensions, lower income, and proportionally greater co-payments and out-of-pocket costs) have been previously identified as factors negatively impacting quality and access to rural health care [16]. Transferring patients with wound category III from rural clinics to urban clinics could be a solution for lack of RIG at rural clinics. Alternative approaches–such as rapidly transporting RIG from the CDC clinic to a rural clinic by a county or private vehicle when needed–could be explored to improve care and minimize burden for patients at rural township clinics. Targeted programs to strengthen rural health care (where rabies virus exposures are most common), including supporting staff training [15, 19], could help counties with a large rural population reduce the number of human rabies deaths. Updating the social insurance scheme to include full reimbursement for costs of PEP (and RIG) could also be helpful.

Proper and thorough wound washing for rabies virus exposures can reduce the amount of virus present and minimize the risk of rabies [14], particularly for category III wounds, if RIG is not available. Approximately half of the patients who should have received the disinfection service at the PEP clinic did not, although most patients reported cleaning the wound prior to seeking care. At the same time, all patients, including four patients with category I wounds according to project observers, received PEP. The unnecessary use of PEP can increase health care costs and impact supply [7, 9, 11]. In 2016, staff at the 17 county A clinics administered 6,000 doses of PEP. This reflects a 13% increase from the 5,272 doses administered by the same clinics in 2015. Similar increases have been identified in other countries, including the Philippines [6] and Tanzania [5], primarily due to increased community awareness, leading to shortages and growing programs costs. The World Health Organization and the Advisory Committee on Immunization Practices in the United States recommend a reduced vaccine schedule for rabies PEP, providing a single dose of rabies vaccine on days 0, 3, 7 and 14 [20, 21]. This reduced schedule can positively impact the rabies vaccine supply and lower the costs to patients.

Although appropriate use of PEP can prevent human deaths, PEP alone does not reduce the risk of subsequent rabies virus exposures [2, 11]. In addition to increasing dog rabies vaccination coverage, investigation of animal bites can provide information to support administration of PEP and help reduce rabies among the dog population [22]. An IBCM approach in Hunan Province could include increasing laboratory capacity to diagnose animal rabies (including introducing sensitive and specific point of care tests for laboratory diagnosis), training of animal surveillance officers and bite investigators, along with the use of equipment for safe animal capture [11, 22, 23]. Following a report of a patient seeking care for an animal wound in county A, for example, investigators could locate and either euthanize the animal for testing or confine the animal for observation [19]. Reports of suspect rabid and dead animals in the community could also be reported and investigated. This approach could be particularly helpful if clinic staff are pressured to administer PEP even when not warranted due to a category I wound or if rabies can be ruled out by the animal passing the 10-day quarantine period [2, 10]. This type of risk-based approach is feasible in Hunan Province as well as other locations that have not yet achieved rabies vaccination coverage of 70% in the dog population [10, 11]. Although the approach has been determined to be cost-effective in Haiti, additional operational research is needed to develop best practices in other rabies endemic countries [10].

The global health community has established a goal of zero dog-mediated human rabies by 2030 [1]. Due to increased awareness and access to PEP, the number of human rabies deaths in China has decreased substantially since peaking in 2007 [19]. Additional work is needed to ensure the thorough investigation of human animal wounds by collecting information on the health and vaccination status of the animal as well as by fully engaging the various animal health sectors for follow-up on suspect rapid and dead animals [2, 11]. Improving diagnostic testing for rabies [22] and implementing dog vaccination campaigns [24] are also needed. Although these steps are important components of rabies elimination, this project highlights the importance of ensuring access and quality delivery of rabies PEP and RIG following possible exposures [23].

Findings from our project were used to develop and conduct a PEP training for clinic staff in Hunan province in May 2018. Training materials and video recordings of the training sessions have been shared with other provinces in China. We are currently reviewing the feasibility of formalizing regular supervisory visits to PEP clinics in Hunan Province to provide supportive guidance and oversight on the appropriate and timely use of PEP. These visits could be conducted in-person or more frequently using online virtual platforms such as WeChat (https://www.wechat.com/en/), Zoom, or other video conferencing software. Although improving appropriate PEP use is important, eliminating dog-mediated human rabies will ultimately depend on country’s ability to vaccinate 70% of the dog population. In China, the Ministry of Agriculture is responsible for licensing and vaccinating the dog population. As with IBCM, the health sector should collaborate closely with the national and provincial level agriculture sectors to coordinate and implement mass dog rabies vaccination campaigns.

Our survey and observational assessment were subject to a few limitations. Firstly, we assessed rabies clinic practices through onsite observation. This may have led to bias as clinic staff may have modified their behaviors for better performance due to being observed. We selected this approach primarily because patient records at rabies PEP clinics do not typically include detailed wound descriptions and information on wound washing and disinfection. Consequently, we were unable to retrospectively verify the wound categories assigned and treatments provided by clinic staff. Second, we assumed that the wound categories assigned by project observers were accurate according to the national guidelines. Although we were unable to assess possible bias resulting from these observations, better health care practices at the urban CDC clinic compared to rural township clinics is consistent with findings from other countries [6, 8, 10, 11]. Third, we visited five PEP clinics selected through purposive sampling. Although these five clinics are similar to other clinics in the county, our findings might not be representative of other clinics in the province. Despite these limitations, our project identified gaps that can be addressed as part of China’s efforts to eliminate dog-mediated human rabies. We developed a training program based on the findings from our project. These trainings, which focused on assigning wound category and administration of PEP, targeted county level staff in Hunan Province. Educational videos were also developed and distributed to rabies clinics in other high-risk provinces in China.

## Conclusion

Clinic staff in county A are providing services to patients following possible exposures to rabid animals. Although we could not rule out possible observer bias, the majority of patients likely received PEP and RIG as recommended. Nevertheless, routine training for staff on wound care, wound categorization and increasing access to RIG, particularly at rural-based clinics, could help improve patient care. Strengthening documentation of wound characteristics and PEP use is also needed. Supportive supervisory visits could help promote and reinforce training goals aimed at improving rabies PEP service delivery. Additional approaches for evaluating human rabies virus exposures, such as IBCM, are feasible and could contribute to China’s progress towards the elimination of dog-mediated rabies.

## Supporting information

S1 File Protocol for the Assessment of Rabies Post-exposure Prophylaxis (PEP) at PEP Clinics in Hunan Province. (DOCX)

S2 File Patient evaluation results after receiving PEP and/or prior to RIG administration. (DOCX)

S1 Table Wound categories and recommended post-exposure prophylaxis (PEP) therapy according to National Guidelines for Human Rabies Control and Prevention, China, 2016. (DOCX)

S2 Table Comparison of clinics selected and not selected for the observational assessment among the 16 rural rabies township clinics in the project county A, Hunan Province, China, 2016. (DOCX)
