# Supplementary material for: Assessing clinicians’ Post-Exposure Prophylaxis recommendations for rabies virus exposures in Hunan Province, China
Source: PLoS Negl Trop Dis. 2021 Jul 6;15(7):e0009564. doi: 10.1371/journal.pntd.0009564 (PMC8284641; doi:10.1371/journal.pntd.0009564)
Supplement: S1 File — (DOCX) [file pntd.0009564.s001.docx]

**Protocol for the Assessment of Rabies Post-exposure Prophylaxis (PEP) at PEP Clinics in Hunan Province**

1. **Overview**

**Title**

A pilot assessment of Post-Exposure Prophylaxis (PEP) practices in Hunan Province, China, 2016.

**Project summary**

A recent review of data collected from the National Rabies Surveillance System has identified a number of possible gaps in reporting and treating rabies exposed individuals, who are potential human cases of rabies. This project will involve implementing an assessment of PEP practices at point-of-care facilities in Hunan Province, a high incidence of human rabies cases. Information captured from this assessment will be used to improve access to and use of appropriate PEP treatment.

**Investigators**

Li Yu, MD, PhD, PI, Division of Infectious Diseases, China CDC.

Jeanette J. Rainey, PhD, MPH, Co-PI, USCDC-Beijing, Will provide technical assistance to the PI (SEV# 7245). Will have no interaction with study participants.

Huai Yang, MD, MPH, Co-PI, USCDC-Beijing. Will provide onsite technical assistance to the PI. Will have no interaction with study participants.

Hu Shixiong, MD, Co-PI, Chief of Epidemiology Branch, Hunan Province CDC.

Miriam Shiferaw, MD, MPH USCDC, Co-PI, Atlanta, USA. Will provide onsite technical assistance to the PI. Will have no interaction with study participants.

OMB/PRA: CDC staff will not approve, direct, manage or own the data collected from the project. All project activities will be conducted by the Principal Investigator and their Chinese staff. Sponsoring Institution: Direct project costs are funded by U.S. CDC Cooperative Agreement #5U2GGH000961-03.

1. **Introduction/Background**

Rabies is a deadly disease with a case-fatality risk approaching 100%. Around 3.9 billion people across 150 countries are at-risk, with approximately 55,000 human deaths due to the rabies reported each year [1-6]. Though canine rabies has been controlled or eliminated for decades in Malaysia, Japan and many island countries in Asia, [7], the zoonotic disease remains endemic in China [8]. A resurgence of rabies has recently occurred in China, peaking in 2007 with 3,300 deaths. The resurgence has primarily affected rural populations in Guizhou, Guangxi, Hunan, Guangdong, and Yunnan Provinces following bites from rabid dogs [8]. Though the incidence has recently declined, the elimination of human rabies remains a public health priority in China.

Rabies post-exposure prophylaxis (PEP) is highly effective at preventing rabies if administered promptly and appropriately after an exposure has occurred. Given that canine surveillance and rabies vaccination programs are still weak in China, PEP is currently the most widely available method for preventing human rabies. Previous studies, however, have suggested that some treated rabies cases were wrongly categorized as exposure I (i.e., touching or feeding animals, licks on intact skin, contact of intact skin with secretions or excretions of a rabid animal or human, and no post-exposure prophylaxis is required) [9]. At the same time, a survey in southeast China indicated that a small proportion of correctly categorized exposed individuals did not receive standard PEP therapy [10]. These observed gaps could be due to issues related to service availability, financial reimbursement, as well as knowledge about PEP therapy by point-of-care staff.

In this project, we propose conducting an assessment of PEP point-of-care facilities to better understand the knowledge, availability to and costs involved with the appropriate administration of PEP. The assessment will target PEP clinic staff in Hunan Province, a high-risk province for human rabies with a relative high rabies incidence. In addition to piloting an assessment tool at selected PEP clinics, all records about patient registration, wound treatment, vaccination and follow up will be reviewed to document PEP administration from January 1 to December 31, 2015). Results from the assessment will be used to identify gaps in diagnosing and treating rabies exposed individuals and better targeting follow-up education and intervention programs, including to PEP clinic staff.

Specific project objectives are outlined below:

1. Develop a set of assessment tools used to assess rabies PEP clinic and rabies related medical practices among clinic staff.
2. Estimate the proportion of rabies exposed persons appropriately classified and treated according to national PEP guidelines.
3. Identify specific gaps in PEP knowledge and practices that can be addressed in future training courses.

**III. Methods**

Definitions

*Human Rabies Case:*

In 2008, the Ministry of Health (MOH) China classified human rabies according to their clinical presentation into two forms: furious rabies and paralytic rabies. Patients diagnosed with either furious or paralytic rabies are defined as probable cases. With epidemiological history, patient either of furious rabies or paralytic rabies was defined as a probable case-patient. Symptoms of furious rabies were clinical symptoms of prickling or itching sensation at the site of bite, progressing within days to agitation, anxiety, confusion, hydrophobia, aerophobia, and paralysis of muscles or cranial nerves, while paralytic rabies without exhibit signs of hyperactivity or hydrophobia, starting at the site of the bite or scratch, muscles gradually become paralyzed, progressing with the systemic flaccid paralysis. The epidemiological history refers to exposure to rabies host animals such as dogs, cats and bats. Probable rabies patients with rabies virus infection detected by direct fluorescent antibody test (DFA), reverse-transcriptase polymerase chain reaction (RT-PCR), or by virus isolation testing of clinical specimens are defined as laboratory confirmed cases.

*PEP:*

In early 2016, China CDC issued guidelines for rabies PEP practice. The guidelines specify that after exposure to suspected infectious sources of rabies PEP must be instituted as soon as possible according to exposure categorization, as defined below.

*Category I*: touching or feeding animals, licks on intact skin, contact of intact skin with secretions or excretions of a rabid animal or human. These are not regarded as exposures, and no post-exposure prophylaxis is required.

*Category II*: nibbling of uncovered skin, minor scratches or abrasions without bleeding. Subjects are to receive local wound treatment and rabies vaccination. Rabies vaccine should be administered as soon as possible. There are two kinds of regimen available for rabies vaccine in China, with one to be 5 dose regimens respectively given at day 0, 3, 7, 14, 28 and the other one to be 2-1-1 regimen given at day 0 (2 doses), 7 (1 dose) and 21 (1 dose) through intramuscular route.

*Category III*: single or multiple transdermal bites or scratches, licks on broken skin, contamination of mucous membrane with saliva from licks and exposure to bats. In addition to wound treatment, rabies vaccine (the same as previously) and immunoglobulin should be administered as soon as possible. As much as anatomically feasible of the immunoglobulin should be infiltrated around the wound at one time, and remainder if any should be injected at an intramuscular site distant from that of vaccine inoculation e.g., into the anterior thigh. The volume of immunoglobulin needed can be determined by 20 IU/ kg for Human RIG (HRIG) or 40 IU/ kg of Equine RIG (ERIG).

PEP clinic recruitment

Shuang Feng County of Hunan Province is proposed as assessment location. Shuang Feng County has reported 12 human rabies cases from 2006 to 2015. Moreover, Shuang Feng County is located within the Prefecture of Lou Di; one of the three high-risk prefectures for rabies in Hunan Province. The reported case number and case incidence between 2010 and 2015 is presented in Table 1. There are numerous rabies PEP clinics in Shuang Feng County, with at least one rabies PEP-clinic in each town. Approximately 5,000 PEP treatments are provided annually in Shuang Feng County. Also, staff capacity Shuang Feng County CDC is very strong and they maintain an advanced rabies surveillance unit. Shuang Feng County leadership supports participation in this project.

**Table 1. Rabies case number and incidence in Lou Di prefecture from 2010 to 2015**

| Year | Case number | Case incidence (1/100,000) |
| --- | --- | --- |
| 2010 | 8 | 0.21 |
| 2011 | 12 | 0.32 |
| 2012 | 8 | 0.21 |
| 2013 | 10 | 0.26 |
| 2014 | 4 | 0.10 |
| 2015 | 6 | 0.16 |
| Sum | 48 |  |

At least 3 clinics will be included in this pilot assessment, including a prefecture-level PEP clinic, a county-level PEP clinic and a town-level PEP clinic. The clinics will be randomly selected from the study area. All doctors and nurses of the selected rabies PEP clinics will be included in the assessment. The assessment will be implemented in summer, during which the number of visiting PEP clinics will peak since individuals wear less clothes and have more chances to be exposed, and therefore, could provide more opportunities to observe PEP practice at the project clinics.

Assessment tools

The assessment tool will aim to capture the quantitative and qualitative information outlined below.

1. Basic descriptive data about the PEP clinic, including clinic affiliation, service hours, services provided (wound treatment and immunoglobulin), type and number of staff members, rabies PEP related equipment and drugs, and regulation as well as management of PEP practices (Appendix 1 - Environment Scan Form of PEP Clinics).
2. Demographic and exposure information about exposed individuals visiting the PEP clinic. Additionally, data on how their exposure is categorized and subsequent practices (wound treatment practice, vaccination, and injection of immunoglobulin as well as adverse events will be recorded) (Appendix 2 - Observation Record Form for Exposure Categorization and PEP).

The assessment will be tablet-based and will be administered by the trained staff members from local CDCs. The assessment will take place at PEP clinics and will aim to involve all clinic staff. An assessment of each clinic will require approximately one week, during which trained staff will assess the clinic through staff interviews, observation of PEP practices, and a medical record review. The records of patients in the past year will be reviewed, in term of the number of PEP subjects, their exposure categorization, and corresponding treatment at the clinic, etc. Our assessment tool will be pilot tested before the official use.

Analysis

The data (the questionnaire of appendix 2) will be collected though a tablet and the data collection software is designed by the IT department of China CDC. China CDC-Beijing will perform the necessary data cleaning and analysis. Collected information will be assessed against the national rabies PEP recommendations. The major outcomes include the proportion of correct exposure categorization and corresponding PEP treatment, including would treatment, vaccination, administration of immunoglobulin, and response to adverse effects of vaccine and immunoglobulin. Chi-square tests will be generated and an alpha level of 0.05 will be used to evaluate statistical significance. Qualitative analysis will involve identification of major themes and outliers.

**IV. Human Subjects Research/IRB Determination**

This project will rely on the analysis of de-identified data for a public health evaluation. Verbal consent will be obtained from participating clinic staff. No personal identifying information will be recorded in the assessment tool. China CDC IRB will review and approve the project protocol prior to implementation. We will also request human subjects research determination by the US CDC.

**V. Expected Outcomes**

We anticipate the following project outcomes:

1. Information on the strengths and gaps in the implementation of PEP practice (risk assessment and subsequent treatment) as recommended by national guidelines.
2. Information on important knowledge gaps that can be addressed in training course planned for health care workers the following year.
3. Feedback on the pilot assessment tool that can be used to improve the tools for similar assessments at other PEP clinics in the future.

**VI. Timeline**

The major project activities are identified below along with the proposed timeline.

- Development of draft assessment tool: May-June 2016
- Pilot test the assessment tool: June 2016
- Conduct assessment: July – August 2016
- Data cleaning and analysis: August - September 2016
- Report preparation: September 2016

**VII. References**

1. Munang’andu HM, Mweene AS, Siamudaala V, Muma JB, Matandiko W. Rabies Status in Zambia for the Period 1985–2004. *Zoonoses Public Health* 2011;58:21–27.

1. Weyera J, Szmyd-Potapczuk AV, Blumberg LH, Leman PA, Markotter W, Swanepoel R, Paweska JT, Nel LH. Epidemiology of human rabies in South Africa, 1983-2007. *Virus Research* 2011;155:283-290.
2. M. HOSSAIN, K. AHMED, T. BULBUL, S. HOSSAIN, A. RAHMAN, M. N. U. BISWAS, A. NISHIZONO. Human rabies in rural Bangladesh. *Epidemiol Infect* 2013;140:1964-–1971.
3. Wang X, Ding S, Li Z, Wang L, Kou Z, et al. Human rabies Epidemiology in Shandong Province, China. *Jpn J Infect Dis* 2010; 63:323-326.
4. Knobel DL, et al. Re-evaluating the burden of rabies in Africa and Asia. Bulletin of the World Health Organization 2005; 83: 360–368.
5. Hossain M, et al. Five-year (January 2004–December 2008) surveillance on animal bite and rabies vaccine utilization in the Infectious Disease Hospital, Dhaka, Bangladesh. *Vaccine* 2011; 29:1036–1040.
6. Hampson K, et al. Rabies exposures, post-exposure prophylaxis and deaths in a region of endemic canine rabies. PLoS Neglected Tropical Diseases 2008;2: e339.
7. Tenzin,M. P. Ward. Review of Rabies Epidemiology and Control in South, South East and East Asia: Past, Present and Prospects for Elimination. Zoonoses and Public Health 2013;59:451–467.
8. [Song M](http://www.ncbi.nlm.nih.gov/pubmed/?term=Song%20M%5BAuthor%5D&cauthor=true&cauthor_uid=24742224), [Tang Q](http://www.ncbi.nlm.nih.gov/pubmed/?term=Tang%20Q%5BAuthor%5D&cauthor=true&cauthor_uid=24742224), [Rayner S](http://www.ncbi.nlm.nih.gov/pubmed/?term=Rayner%20S%5BAuthor%5D&cauthor=true&cauthor_uid=24742224), et al. Human rabies surveillance and control in China, 2005-2012. BMC Infect Dis. 2014,14:212.
9. Song M, Tang Q, Wang DM, et al. Epidemiological investigations of human rabies in China. BMC Infectious Diseases, 2009, 9:210.
10. Zhu KR, Yue Y, OU Z, et al. Medical Staff's Capability of Treating Dog-bitten Out-patients in Rabies Clinics in Chengdu. J Prev Med Inf, 2013, 29(2): 99-102.

**VIII. Appendices**

1. Environment Scan Form of PEP Clinics
2. Observation Record Form for Exposure Categorization and PEP

**Appendix 1**

**Environment Scan Form of PEP Clinics**

**General information**

1. Address: province prefecture town street/village____________________________________________________
2. Is the clinics issued the Practicing License of Medical Institution? □ Yes □ No
3. Service time:

□ 7 days per week, 24 hours for a day

□ week days □□:□□——□□:□□, no staff on duty by phone for the rest

□ week days □□:□□——□□:□□, staff on duty by phone for the rest, is there phone number publicly displayed at prominent place: □ Yes □ No

□ Other

1. How to operate the PEP clinic?

□ Independently

□ Affiliated to vaccination clinic

□ Affiliated to medical establishment

□ Other

4.1 If it is affiliated to other clinic or medical establishment, is it separated with other services in spatial terms: □ Yes □ No

1. Is there specific area for wound treatment? □ Yes □ No
2. Is the clinic entitled to surgery therapy qualification? □ Yes □ No

6.1 If no, is there any surgeon or general practitioner join the complicated wound treatment nearby? □ Yes □ No

**Staff setting**

7. Basic information of staff in clinic

| ID | Name | Gender | Age | Education^a^ | Qualification^b^ | Profession^c^ | Training verification^d^ | Allergy treatment training^e^ | Received rabies vaccination |
| --- | --- | --- | --- | --- | --- | --- | --- | --- | --- |
|  |  |  |  |  |  |  |  |  |  |
|  |  |  |  |  |  |  |  |  |  |
|  |  |  |  |  |  |  |  |  |  |
|  |  |  |  |  |  |  |  |  |  |
|  |  |  |  |  |  |  |  |  |  |

a) Education: 1.Postgraduate 2.College 3.Junior college 4.High school

b) Qualification: 1. Licensed doctor 2. Licensed assistant doctor 3. Nurse 4. Rural doctor 5. Other

c) Profession: 1. Public health 2. Disease control 3. General practice 4. Surgery 5. Other

d) Received the PEP training organized by county health administrative department or above, and passed it? 1. Yes 2. No

e) Received emergency treatment training for allergy caused by vaccine? 1. Yes 2. No

f) Received rabies vaccination? 1. Yes 2. No

8. Have the staffs been organized to receive rabies vaccination?

□ Yes, frequency: □ Semi-annually □ Annually □ Biyearly □ Other

□ No

**Biological product**

9. The kind of vaccine in clinic: □ “2-1-2” vaccine, Persons in stock

□ Vaccine for 5 doses regime, Persons in stock

10. The kind of passive immunity preparation:

□Human Rabies Immunoglobulin, IU in stock

□ Rabies antiserum, IU in stock

11. Purchase source of rabies vaccine in clinic (multiple choices)

□ County CDC □ Prefecture CDC □ Provincial CDC

□ Company □ Other________________________________________

12. Purchase source of passive immunity preparation (multiple choices)

□ County CDC □ Prefecture CDC □ Provincial CDC

□ Company □ Other________________________________________

**Facility and equipment**

13. Is the clinic equipped with professional equipment for wound flushing? □ Yes □ No

14. Is there any wound rinse?

□ Yes □Soap □Suds □Other

□ No

15. Is there any sterilize

□ Yes □Iodine □Benzalkonium chloride □Other

□ No

16. Is there any equipment in clinic as below?

□ Fridge □ Thermometer (to monitor the temperature in fridge) □ Weighing scale □Thermometer (to measure human body)

17. Are there rescue equipment and drugs for allergy?

□ Rescue bedding □ Oxygen supply □ Simple sambal □Anesthetic laryngopharyngoscope

□ Hematomanometer □Stethoscope □ Epinephrine □ Promethazine □ Dexamethasone

□ Cedilanid □ Lobeline □ Metaraminol □ Nikethamide □ Glucose injection

□ Other

17.1 If there is no rescue equipment or drugs, how to deal with severe allergy?

**Information management**

18. Were the informed consents signed by exposure subjects in clinic saved?

□ Yes □ No

19. Are there any records on exposed subjects in the past year (include basic information, wound category, following disposal) ?

□ Yes, in the form of electronic? □ Yes □ No

□ No

20. The summary of the records on exposed subjects from 2013/1/1-2015/12/31.

| Number of outpatient | Exposure degree | | | Treatment of wound | | | Vaccination | | | Injection of passive immune agents | Combined | |
| --- | --- | --- | --- | --- | --- | --- | --- | --- | --- | --- | --- | --- |
|  | Degree Ⅰ | Degree Ⅱ | Degree Ⅲ | Treatment by oneself | Treatment by outpatient department | No treatment | Whole course | No whole course | No vaccination |  | No whole course | Whole course |
|  |  |  |  |  |  |  |  |  |  |  |  |  |

Assessor：_________________ Date of assessment：□□(day)/□□(month)/□□□□(year)

**Appendix 2**

**Evaluation Form for Rabies Exposure Prevention and Treatment**

Outpatient Number __________ Patient number __________

Age of exposed patient __________ Sex of exposed patient ①Male ② Female

**Inquiry stage**

1. Did the staff ask the exposed person about the following conditions that injured the animal:

1.1 Species: ①dog; ②cat; ③other__________；④not asked

1.2 Source: ①own house; ②other house; ③stray animals; ④wild animals; ⑤unsure; ⑥not asked

2. Did the staff ask the exposed person about the exposure time?

①Yes; ②No (skip to 3)

2.1 Exposure time: __________ year __________ month __________ day __________ hour

3. Did the staff ask and check the exposed parts of the exposed person?

①Yes; ②No

3.1 Exposure parts (multiple choices): ①head and face; ②neck; ③trunk; ④left arm; ⑤right arm; ⑥left hand; ⑦Right hand; ⑧Left lower limb; ⑨Right lower limb; ⑩Other __________

3.2 Exposure details (subject to the most severe wound)

①Contact or feed animals

②Intact skin is licked

③Bare bite on bare skin

④Slight scratch or abrasion without bleeding

⑤Single or multiple penetrating skin bites or scratches

⑥Broken skin is licked

⑦Open wound or mucous membrane is contaminated

⑧Others (please describe in detail): __________

3.2.1 If the exposed person sees a doctor in time (that is, within 3-4 hours after exposure), whether the exposed skin is difficult to judge with the naked eye, carry out a skin damage test:

①Yes; the results of the skin damage test are: ①positive (rubbing alcohol is painful); ②negative (rubbing alcohol is not painful)

②No

3.3.1 Whether to record the number of wounds: ①Yes; ②No

3.3.2 Number of wounds: __________ places

3.4.1 Whether to record the size of the wound:

①Yes; ②No ③No need to record

3.4.2 The largest wound size: __________ cm × __________ cm

3.5.1 Whether to record the depth of the wound:

①Yes; ②No ③No need to record

3.5.2 The deepest part of the wound: __________ mm

4. Whether to ask the exposed person to clean the wound by himself after being injured:

□ Not asked

□ Asked, as follows:

①Rinse with clean water, duration: __________ minutes

②Rinse with detergent, duration: __________ minutes

③Clear water and detergent are applied alternately, duration: __________ minutes

④Not rinsed

⑤Did not ask about the specific situation

5. Whether to ask the exposed person about self-disinfecting wounds after being injured:

□Not asked

□Inquired, as follows:

①Iodine disinfection; ②Alcohol disinfection; ③No disinfection; ④Other__________;⑤No specific information was asked.

6. The outpatient staff judges the exposure classification as:

①level I; ②level II; ③level III

7. Whether to ask the exposed person about the history of rabies vaccination:

①Never vaccinated; ②Vaccination, but not the whole course of vaccination; ③Vaccination, but did not ask whether the whole course was vaccinated; ④Vaccination, but did not ask the time of vaccination; ⑤The whole course of vaccination, the completion time is __________ year __________ month; ⑥Not asked

8. Whether to ask the exposed person for the history of diseases related to low immunity (HIV/AIDS, tuberculosis, malignant tumor, other basic/chronic diseases)?

①Yes, □Yes □No; ②No

9. Whether to ask the drug allergy history of the exposed person?

①Yes, □Yes □No; ②No

10. Whether to ask about the use of drugs such as immunosuppressants and glucocorticoids? (Such as dexamethasone, cortisone, and prednisone)

①Yes, □Yes □No; ②No

11. Are the exposed persons or their family members required to sign the informed consent form (regardless of whether they are vaccinated and/or passive immunization agents)?

①Yes; ②No

**Wound treatment stage**

1. Did the medical staff wash the wound for the exposed person?

①Yes; (Jump 2) ②No, the reasons are as follows:

□The wound has scabbled or healed (skip to post-exposure immunity)

□Medical staff need to wash the exposed persons or accompanying persons themselves (skip to 4)

□The exposed person thinks that washing is unnecessary (skip to post-exposure immunity)

□Flushing in other medical institutions (skip to post-exposure immunity)

□The exposed person has washed the wound by himself (skip to post-exposure immunity)

□Other __________

2. Whether medical staff wear work clothes: ①Yes; ②No

3. Whether medical staff disinfect their hands or wear gloves: ①Yes; ②No

4. Wound irrigation

4.1 Clean water flow used for flushing:

①Pressure flowing water such as tap water; ②Pressure-free flowing water poured with a ladle; ③Wash directly in the basin; ④Others: __________

4.2 Whether to use detergent: ①Yes; ②No;

4.3 Types of cleaning agents:

①solid soap; ②semi-solid soap (soft soap) ③liquid detergent (such as soapy water, hand sanitizer, etc.); ④commercial dog wound cleaner; ⑤other__________

4.4 Are the cleaning agents used for cleaning shared with other exposed persons? ①Yes; ②No

4.5 Rinse time: __________ minutes

4.6 Whether to alternately use clean water and detergent to treat wounds: ①Yes; ②No

4.7 Whether to use sterile absorbent cotton to absorb the residual fluid at the wound: ①Yes; ②No

5. Do medical staff disinfect the wounds of exposed persons?

①Yes; (skip to 5.1); ②No, the reasons are as follows:

□The wound has scabbled or healed (skip to post-exposure immunity)

□Medical staff should disinfect themselves by themselves (skip to post-exposure immunization)

□Medical staff believe that disinfection is not necessary (skip to post-exposure immunity)

□The exposed person thinks that disinfection is not necessary (skip to post-exposure immunity)

□ have been disinfected in other medical institutions (skip to post-exposure immunization)

□The exposed person has disinfected the wound by himself (skip to post-exposure immunity)

□Other __________ (skip to post-exposure immunity)

5.1 Name of disinfectant (please specify concentration and active ingredient): ①2-3% iodine (iodine); ②75% alcohol; ③3% hydrogen peroxide; ④others

5.2 The scope of wound disinfection: the wound and the area within __________ cm around it

5.3 Whether the sequence of wound disinfection is to wipe from the center of the wound to all around: ①Yes; ②No

6. Do medical staff suture the wound?

①Yes; the stitching time is □□:□□ (24-hour system)

②No, the reasons are as follows:

□The medical staff thinks that the wound is unnecessary to be stitched

□The wound is crusted or healed

□Exposed strongly request not to suture

□Other__________

**Post-exposure immunity**

1. Are you vaccinated? ①Yes; ②No, the reasons are as follows:

□Medical staff do not recommend vaccination

□The price is too expensive

□The exposed person thinks it is unnecessary

□Other __________

1.1 Inoculation procedure: ① "2-1-1"; ② 5-shot method

1.2 The dose and location of the first vaccination:

①1 dose: □left upper arm deltoid muscle □right upper arm deltoid muscle □left anterolateral thigh muscle □ Right anterolateral thigh muscle □ Other __________

②2 doses: □left upper arm deltoid muscle □right upper arm deltoid muscle □left anterolateral thigh muscle □ Right anterolateral thigh muscle □ Other __________

2. Whether hands are disinfected, or gloves are worn during vaccination:

①Yes; ②No

3. Do you wear work clothes during vaccination? ①Yes; ②No

4. Whether the vaccine is within the validity period: ①Yes; ②No

5. Rabies freeze-dried vaccination:

5.1 Whether to bomb the tip of the ampoule to the bottom: ①Yes; ②No

5.2 Whether to use 75% ethanol cotton ball or iodine tincture to disinfect the ampoule: ①Yes; ②No

5.3 Whether to shake well after adding diluent: ①Yes; ②No

5.4 Whether to empty the bubbles in the syringe: ①Yes; ②No

5.5 After the syringe draws the vaccine, whether the needle is emptied in an ampoule, and placed in a sterile towel for use: ①Yes; ②No；③No need, direct injection.

5.6 Whether to withdraw the syringe: ①Yes; ②No

5.7 Whether to press after the needle is pulled out: ①Yes; ②No

5.8 Whether the hands of medical staff are disinfected after vaccination:

①Yes; ②No

6. Is the vaccinated person required to stay in the outpatient clinic after the first vaccination?

①Yes; Observe for half an hour or more; Observe within half an hour; ②No

7. After the first vaccination, have the exposed persons informed of the possible abnormal vaccine reactions and treatment methods:

①Yes; ②No

8. Are the passive immunization preparations and rabies vaccine injected at the same site? ①Yes; ②No; ③Not applicable

9. Is the same syringe used for vaccination and passive immunization preparations? ①Yes; ②No; ③Not applicable

10. Do you sign the disposal sheet and fill in the details of the injection (batch number, expiration date, etc.) and execution time? ①Yes; ②No；③No disposal sheet

**Passive immune preparation injection**

1. Whether to inoculate passive immunity preparations? (Single choice)

①Yes, the time to complete the injection is □□:□□ (24-hour system);

②No, the reasons are as follows:

□Medical staff do not recommend vaccination of passive immunization preparations (skip to reviewer’s signature)

□The price is too expensive (skip to the reviewer’s signature)

□The exposed person thinks it is unnecessary (skip to the reviewer's signature)

□Other__________ (skip to reviewer’s signature)

2. Whether to ask or measure your weight? ①Yes __________ Kg; ②No

3. The injection dose prescribed by the doctor is __________ each __________ IU)

4. Check whether the injection information (such as name, gender, age, etc.) is consistent with the prescribed immunoglobulin before injection: ①Yes; ②No

5. Do medical staff disinfect their hands or wear gloves before vaccination: ①Yes; ②No

6. Preparation for rabies immune globulin injection:

6.1 Whether to use 75% ethanol cotton ball to disinfect the medicine bottle: ①Yes; ②No

6.2 Whether the disinfectant is clearly marked (variety, concentration, validity period, activation time): ①Yes; ②No

6.3 Whether to inhale the required amount of air of the medicine/dispensing liquid in the syringe: ①Yes; ②No

6.4 Whether the needle is in contact with the surface of other objects during this process: ①Yes; ②No

6.5 Whether to invert the medicine bottle and pull the syringe needle to the correct dose

6.6 Whether to confirm again that sufficient amount of medicine has been drawn: ①Yes; ②No

6.7 Whether to flick the syringe barrel to remove the residual bubbles in the syringe to expel air bubbles: ①Yes; ②No

6.8 If the syringe needs to be capped after the immunoglobulin is absorbed, whether to use one-handed capping. ①Yes; ②No

6.9 If you need to spare, whether to put the capped syringe into the tray in the sterile towel for use: ①Yes; ②No

7. Anti-rabies immune globulin injection

7.1 Exposed parts, limbs, trunk, etc.

7.1.1 After the wound has been treated, do you put a few drops in the wound HRIG: ①Yes; ②No

7.1.2 Needle insertion position: ①At __________ cm from the wound edge; ②Wound site ③Other__________

7.1.3 Needle entry angle: __________ degree angle

7.1.4 Whether the needle is inserted to the base of the wound: ①Yes; ②No; ③Unable to judge

7.1.5 Whether to withdraw the needle while pushing the liquid medicine: ①Yes; ②No

7.1.6 Whether to change the direction while withdrawing the needle, and inject at 12 o'clock and 6 directions at the edge of the wound (take the corresponding two points in the vertical and left and right directions for circular full-thickness injection): ①Yes;

7.1.7 Whether the wound is withdrawn first before the injection of the drug: ①All withdrawn; ②Partial withdrawn ③Not withdrawn

7.1.8 Whether a three-dimensional seal is formed at the bottom of the wound: ①Yes; ②No③Can't judge

7.1.9 Does the injected HRIG leak from the wound: ①Yes; ②No

7.1.10 After all wounds are infiltrated and injected, the remaining HRIG injection sites: ①Ipsilateral back muscles of the exposed part; ②Exposed lateral thigh muscles of the same side ③Other__________

7.2. Exposure of head, face, neck, etc.

7.2.1 Whether to use a syringe to drip HRIG into the wound: ①Yes; ②No ③Other

7.2.2 Remaining HRIG injection sites: ①Ipsilateral back muscles at the exposed site; ②Exposed lateral thigh muscles at the same side ③Other

7.2.3 Whether to withdraw the remaining HRIG before injecting and pushing: ①All withdraw; ②Partial withdraw ③No withdrawal

7.3 Exposure of conjunctiva, oral cavity, nasal mucosa, etc.

7.3.1 Whether to drip or smear HRIG on the mucous membrane several times: ①Yes; ②No

7.3.2 Remaining HRIG injection sites: ①Ipsilateral back muscles at the exposed site; ②Exposed lateral thigh muscles at the same side ③Others

7.3.3 Whether to withdraw the remaining HRIG before injection and push: ①All withdraw; ②Partial withdraw ③No withdrawal

7.4 Exposure of testes, penis, anus, etc.

7.4.1 Put a vial containing an appropriate amount of HRIG into the wound for a while: ①Yes; ②No

7.4.2 The remaining HRIG is injected intramuscularly into the anterolateral thigh: ①Yes; ②No

7.4.3 Whether to withdraw the remaining HRIG before injecting and pushing: ①Withdraw all; ②Partial withdraw ③Without withdrawal

7.5 Exposure of hands and feet

7.5.1 Drip HRIG around the wound for spot infiltration injection: ①Yes; ②No

7.5.2 Whether the injected HRIG leaks from the wound: ①Yes; ②No

7.5.3 Wound infiltration must be withdrawn before injection: ①All withdraw; ②Partial withdraw ③Without withdrawal

7.5.4 Remaining HRIG injection sites: ①Ipsilateral back muscles at the exposed site; ②Exposed lateral thigh muscles at the same side

7.5.5 The remaining HRIG must be withdrawn before injecting and pushing: ①All withdraw; ②Partial withdraw ③No withdrawal

8. Whether to press after needle removal: ①Yes; ②No

**Reviewer:** __________

**Review date:** __________ **year** __________ **month** __________ **day** __________ **hour**
