# Supplementary material for: Assessing clinicians’ Post-Exposure Prophylaxis recommendations for rabies virus exposures in Hunan Province, China
Source: PLoS Negl Trop Dis. 2021 Jul 6;15(7):e0009564. doi: 10.1371/journal.pntd.0009564 (PMC8284641; doi:10.1371/journal.pntd.0009564)
Supplement: S2 File — (DOCX) [file pntd.0009564.s002.docx]

**S2 File. S4 File. Patient’ evaluation results after receiving PEP and/or prior to RIG administration.**

Of the 195 patients who received PEP, clinic staff only required 109 (56%) to stay for observation for possible adverse reactions. This was required more frequently by staff at the rural clinics (n = 63, 75%) compared to the urban clinic (n = 46, 41%) (χ^2^=20.5, p-value <0.001). Additionally, of the 38 patients receiving RIG, clinic staff asked for, or measured, the weight of only 24 patients (63%) as recommended.
