# Supplementary material for: Assessing clinicians’ Post-Exposure Prophylaxis recommendations for rabies virus exposures in Hunan Province, China
Source: PLoS Negl Trop Dis. 2021 Jul 6;15(7):e0009564. doi: 10.1371/journal.pntd.0009564 (PMC8284641; doi:10.1371/journal.pntd.0009564)
Supplement: S1 Table — (DOCX) [file pntd.0009564.s003.docx]

**S1 Table. Wound categories and recommended post-exposure prophylaxis (PEP) therapy according to National Guidelines for Human Rabies Control and Prevention, China, 2016.**

| **Wound Category** | **Wound categorization criteria** | **Recommended PEP** |
| --- | --- | --- |
| **I** | Meeting one of the following conditions:  1. Touching or feeding animals  2. Licks on intact skin  3. Contact of intact skin with secretions or excretions of rabid animal or human case | 1. No PEP is required. |
| **II** | Meeting one of the following conditions:  1. Nibbling of uncovered skin  2. Minor scratches or abrasions without bleeding | 1. Wound treatment immediately  2. Rabies vaccination immediately |
| **III** | Meeting one of the following conditions:  1. Single or multiple transdermal bites or scratches  2. Lick on broken skin  3. Contamination of mucous membrane with saliva (such as licking)  4. Exposure to bats | 1. Wound treatment immediately  2. Rabies vaccination^a^ immediately  3. RIG^b^ immediately |

^a^Clinics can use either Zagreb 2-1-1, in which two doses of vaccine are injected intramuscularly on day 0 (one into each of the two deltoid or thigh sites) followed by one of each dose on days 7 and 21, or the five-dose Essen regimen, in which a single dose is administered intramuscularly on days 0, 3, 7, 14, and 28.

^b^Both RIG derived from human blood (hRIG) or equine blood (eRIG) are approved in China.
