# Supplementary material for: Assessing clinicians’ Post-Exposure Prophylaxis recommendations for rabies virus exposures in Hunan Province, China
Source: PLoS Negl Trop Dis. 2021 Jul 6;15(7):e0009564. doi: 10.1371/journal.pntd.0009564 (PMC8284641; doi:10.1371/journal.pntd.0009564)
Supplement: S2 Table — (DOCX) [file pntd.0009564.s004.docx]

**S2 Table. Comparison of the clinics selected and not selected for the observational assessment among the 16 rural rabies township clinics in the project county A, Hunan Province, China, 2016.**

| **Characteristics** | **Selected rural clinics (N=4)** | **Unselected rural clinics (N=12)** |
| --- | --- | --- |
| **Number of patients seeking rabies PEP in 2016** | 1,510 | 1,797 |
| **Service hours of clinic, n (%)** |  |  |
| **24/7** | 3 (75) | 11 (92) |
| **Specific time of every day** | 1 (25) | 1 (8) |
| **Staff number per clinic, median [range]** | 3 [2-5] | 4.5 [1-7] |
| **Highest education level of staff, n (%)** |  |  |
| **College** | 1 (8) | 1 (2) |
| **Junior college** | 8 (62) | 19 (37) |
| **Professional high school** | 4 (31) | 31 (61) |
| **With special area for wound treatment, n (%)** | 2 (50) | 6 (50) |
| **With professional wound washing equipment, n (%)** | 0 (0) | 0 (0) |
| **With refrigerator, n (%)** | 4 (100) | 12 (100) |
| **With rabies immunoglobulin, n (%)** | 0 (0) | 0 (0) |
| **Price in US dollars for vaccine per whole course, median [range]** | 46 [43-48] | 50 [46-61] |
